# Isolated duodenal myeloid sarcoma associated with the *CBFβ*/*MYH11* fusion gene followed by acute myeloid leukemia progression: A case report and literature review

**DOI:** 10.3892/ol.2014.2313

**Published:** 2014-07-04

**Authors:** BO HUANG, PENG YOU, PING ZHU, ZUNGUO DU, BEIQIAN WU, XIAOPING XU, ZI CHEN

**Affiliations:** 1 Department of Hematology, Huashan Hospital, Fudan University, Shanghai 200040, P.R. China; 2 Department of Gastroenterology, People’s Hospital, Peking University, Beijing 100044, P.R. China; 3 Department of Pathology, Huashan Hospital, Fudan University, Shanghai 200040, P.R. China

**Keywords:** *CBFβ/MYH11* fusion gene, acute myeloid leukemia, myeloid sarcoma

## Abstract

Myeloid sarcoma (MS) is a rare disease that presents as an extramedullary tumorous mass of immature myeloid precursors. The majority of MS are identified in acute myeloid leukemia (AML) patients and rarely present as a primary isolated MS without AML. In addition, inversion of chromosome 16 [inv(16)] and the *CBFβ*/*MYH11* fusion gene are rarely associated with MS. The current study reports a female patient with an isolated duodenal MS, who developed AML-M4 associated with the *CBFβ*/*MYH11* fusion gene and 48,XX,inv(16),+13,+22. A review of previously reported cases of isolated MS with the *CBFβ*/*MYH11* fusion gene was also performed. Isolated MS with the *CBFβ*/*MYH11* fusion gene was often observed in abdominal lesions, with the intestinal tract being the predominantly involved site. In addition, patients with isolated MS with the *CBFβ*/*MYH11* fusion exhibited a high risk of developing systemic AML. The diagnosis of isolated MS may be particularly challenging and, therefore, determining the optimal standard treatment for isolated MS is required.

## Introduction

Myeloid sarcoma (MS) is a tumor mass consisting of myeloid blasts with or without maturation occurring at an anatomical site other than the bone marrow (BM). The disease was first described in 1811 by Burns (1). The term ‘granulocytic sarcoma’ was first used by Rappaport in 1966 to describe MS (2) and the World Health Organization (WHO) Classification of Tumours of Haematopoietic and Lymphoid Tissues adopted the use of the term MS in 2008 (3). MS may precede, follow or occur simultaneously with acute myeloid leukemia (AML). MS rarely develops in patients with chronic myeloid leukemia, myelodysplastic syndromes (MDS) or myeloproliferative neoplasms (MPN). Isolated MS is rare and defined by the absence of a history of leukemia, MPN and MDS, as well as a negative BM examination. Furthermore, MS has been associated with various cytogenetic abnormalities. Only a small number of cases with inversion of chromosome 16 [inv(16)] and the *CBFβ*/*MYH11* fusion gene have been reported (4–13). The present study describes a case of an isolated duodenal MS associated with the *CBFβ*/*MYH11* fusion gene followed by AML progression. Patient provided written informed consent.

## Case report

**Figure 1:**
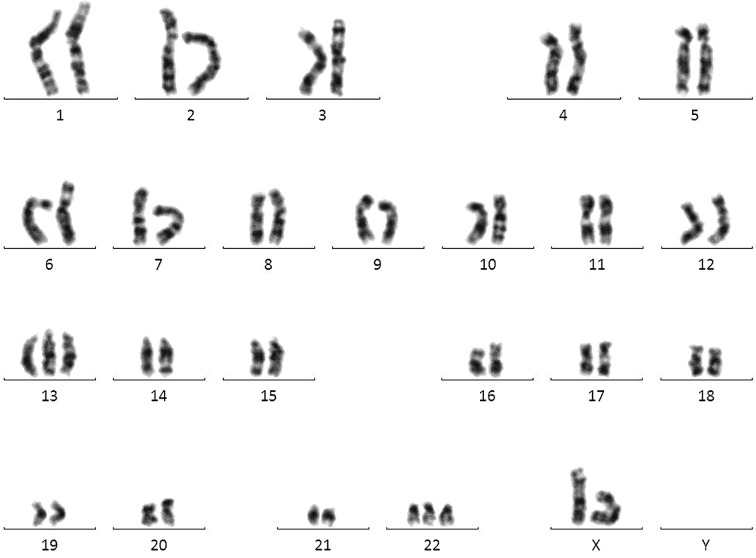
Cytogenetic analysis by G-banding showing 48,XX,inversion of chromosome 16,+13,+22.

A 58-year-old female was admitted to the Department of Gastroenterology at the Huashan Hospital (Shanghai, China) in June, 2013 with the chief complaint of constantly feeling full and abdominal pain for one month. At one week prior to admission, the patient underwent an upper endoscopy (EGD) examination at Tai’an City Hospital (Tai’an, China), which demonstrated a mass in the duodenum. Biopsies of the lesion indicated a lymphoma. On admission to the Huashan Hospital, decreased bowel sounds and tenderness upon palpation in the left upper quadrant were notable during the physical examination, however, no shifting dullness or hepatosplenomegaly were identified. Upper gastrointestinal series indicated incomplete obstruction of the descending duodenum. In addition, computed tomography (CT) of the abdomen showed thickening of the intestinal wall of hte descending duodenum with heterogeneous contrast enhancement. A repeat EGD was performed following admission, which revealed luminal stenosis of the descending duodenum and multiple proliferative lesions that were subsequently biopsied. The biopsy revealed blast cells, which were positive for myeloperoxidase (MPO), cluster of differentiation (CD)34, CD117 and CD43, and negative for CD20, CD3, CD2, CD79, CD5, CD1a and CD56. Whereas, the BM aspiration and biopsy did not show blasts. Laboratory studies, including blood routine, liver function and renal function tests, as well as the partial thromboplastin and prothrombin times, were within normal range. The patient was diagnosed with duodenal MS and subsequently underwent an additional BM aspiration 14 days following the initial BM examination. The results showed hypercellularity with 30% myeloblasts, 23% monoblasts and 2.5% eosinophils. Flow cytometry identified the positive expression of CD34, CD117, CD15, CD33, CD38, HLA-DR, CD13, MPO and CD36, and cytogenetic analysis showed karyotype 48,XX,inv(16),+13,+22 (Fig. 1). Leukemia-associated fusion gene analysis detected positive expression of *CBFβ*/*MYH11*, and negative expression of *BCR-ABL, TEL-ABL, PML-RAR*α*, PLZF-RAR*α*, MLL-AF9, AML-ETO, E2A-PBX1, E2A-HLF, MLL-AF4, MLL-AF6, MLL-AF10, MLL-ENL, MLL-AF17, MLL-AF1q, MLL-ELL, MLL-SEPT6, NPM-RAR*α*, NPM-MLF, AML1-MDS1*/*EVI1, AML1-MTG16, AML1-EAP, FIP1L1-PDGFRA, SET-CAN, DEK-CAN, TEL-AML1, TEL-PDGFRB, TLS-ERG* and *SIL_TAL1*. Based on the abovementioned results, the final diagnosis was AML, subtype M4 [according to the French-American-British (FAB) classification system] associated with the *CBFβ*/*MYH11* fusion gene and duodenal MS. The patient was administered anthracycline (14 mg/m^2^ losomal doxorubicin intravenously on days one and two) and cytarabine (100 mg/m^2^ on days one to five) for one cycle of 28 days. Following the induction of chemotherapy, the abdominal symptoms disappeared and a BM examination showed complete remission (CR) with only 0.5% blasts. The patient then received one further cycle of losomal doxorubicin (28 mg/m^2^ intravenously on day one) and cytarabine (100 mg/m^2^ cytarabine on days one to seven) for one cycle of 28 days, as well as two courses of high-dose cytarabine (2 g/m^2^ on days one, three and five) for one cycle of 28 days for consolidation. A repeat CT scan showed that there was no tumor in the abdomen and a BM examination indicated complete hematologic and molecular remission. At present, the patient is being followed up while undergoing regular chemotherapy and no signs of disease relapse have been observed.

## Discussion

MS is a neoplastic proliferation of immature myeloid precursors forming a tumorous mass, which occurs at anatomical sites other than the BM. The incidence of MS is 2.5–9.1% among patients with AML in Europe and the USA (14–16). MS is most common in certain subtypes of AML, in particular M5, M4 and M2 according to the FAB classification system. Isolated MS, also termed aleukemic, primary or *de novo* MS, is particularly rare. Isolated MS has been described in limited case reports and the incidence of isolated MS remains unclear.

Specific cytogenetic abnormalities and genetic mutations have been reported in MS. The t(8,21) translocation is the most common recurrent chromosomal abnormality associated with MS. Furthermore, the *AML1-ETO* (*RUNX1-RUNX1T1*) fusion gene is consistently detected in patients with the t(8;21) translocation (17,18). An additional common recurrent chromosomal abnormality in MS is inv(16), which is associated with the *CBFβ*/*MYH11* fusion gene. In the majority of cases, MS with these two recurrent cytogenetic abnormalities occur in AML with the same cytogenetic abnormalities; these MS cases are the extramedullary manifestation of AML. In rare cases, isolated MS with a recurrent cytogenetic abnormality may develop.

The current study presents a rare case in which a duodenal isolated MS, which was associated with the *CBFβ*/*MYH11* fusion gene and inv(16), rapidly progressed (within two weeks) to overt AML. A search for similar case reports via the PubMed database retrieved only 10 reported cases of isolated MS involving inv(16) or the *CBFβ*/*MYH11* fusion (4–13). Among these 10 cases, two cases were not included in the current review. One of the two cases (4) was an isolated MS with inv(16) and 10.4% myeloblasts in the BM. According to the recommendation of the WHO (2008), cases with inv(16)(p13.1;q22) and <20% BM blasts must be diagnosed as AML (19). The other case report (5) was MS following an allogeneic hematopoietic stem cell transplantation (Allo-HSCT). Prior to the Allo-HSCT, the patient was diagnosed with AML with inv(16) and based on the information, the MS appeared to be an extramedullary relapse of AML. The remaining eight cases of isolated MS and the current study are listed in Table I (6–13).

Of the nine cases included in this study, the isolated MS with *CBFβ*/*MYH11* fusion and inv(16) was consistently identified in the abdomen (eight out of nine cases). Furthermore, the intestinal tract was the most commonly involved site, including the small intestine and mesentery. It appears that the small intestine is a relatively specific target for MS with the *CBFβ*/*MYH11* fusion. Abdominal pain and/or intestinal obstruction are the most common symptoms.

The diagnosis of MS in the setting of leukemia is not complex. However, in the absence of a history of leukemia, the diagnosis of MS becomes challenging. One study has shown that ~40% of patients with isolated MS are initially diagnosed as other conditions, most often as diffuse large B-cell lymphoma (20,21). The diagnosis is based on morphological examination and immunohistochemistry. The use of specific markers for myeloid disease, such as MPO, CD43, lysozyme, CD33, CD117 and CD34, is required to establish the diagnosis of MS. Other immunohistochemistry markers may be used depending on the differential diagnosis. The differential diagnosis for MS is broad, including non-Hodgkin’s lymphoma, blastic plasmacytoid dendritic cell neoplasm, histiocytic sarcoma and poorly differentiated carcinoma.

Due to the rarity of isolated MS, the optimal treatment has not yet been established. However, it is well known that the delayed or inadequate treatment of isolated MS almost always leads to AML (16). In the current literature review, five of the nine patients with isolated MS developed AML. Although the other four patients did not show overt evidence for AML, AML clones were detected in the BM using cytogenetic and/or molecular biological polymerase chain reaction-based methods.

The current recommended treatment for isolated MS is conventional AML-type chemotherapy protocols. This recommendation is based on the observation of a markedly higher risk of progression to AML in patients with isolated MS receiving localized treatment compared with isolated MS patients receiving systemic chemotherapy (16,22). Anthracycline and cytarabine are the standard remission induction chemotherapy agents and the consolidation treatment regimen is administered following CR as in AML. The CR rate of AML with the *CBFβ*/*MYH11* fusion is reported to be between 81 and 93%, which is markedly higher than the majority of other subtypes of AML (23,24). The prognosis of AML with the *CBFβ*/*MYH11* fusion and inv(16) is optimistic compared with other types of AML, with disease-free survival ranging between 48 and 63% (23,24). Allo-HSCT is therefore not the first-line treatment option and is usually reserved for relapsed or refractory cases. It remains unclear whether similar CR rates and the optimal prognosis of MS with the *CBFβ*/*MYH11* fusion can be predicted based on the current data concerning AML with the *CBFβ*/*MYH11* fusion.

In conclusion, in the present case, the patient responded well to the standard induction therapy followed by the high-dose cytarabine-based consolidation treatment. However, due to the lack of experience regarding the treatment of this rare disease, controlled prospective multicenter clinical trials are considered to be important and necessary to guide treatment.

## Figures and Tables

**Table I:** Nine reported cases of isolated MS associated with the *CBFβ/MYH11*fusion gene and/or inv(16) AML.

Age, years	Gender	Karyotype	Fusion gene detection	MS location	Progression to AML	Reference
49	M	49,XY,inv(16)(p13q;22),+6,+14,+22	NA	Small intesine	Yes	6
30	M	46,XY,inv(16)(p13q;22)	NA	Small intesine	Yes	7
25	M	47,XY,inv(16)(p13;q22),+22	Yes	Mesentery	No	8
37	M	46,XY[3]47,XY,+9,inv(16)(p13;q22)[1] 48,XY,+9,inv(16)(p13;q22),+22[19] 49,XYY,+9,inv(16)(p13;q22),+22[2]	NA	Jejunum	Yes	9
23	F	47,XX,+8, inv(16)(p13;q22)	NA	Breast	Yes	10
50	F	inv(16)(p13;q22)	Yes	Small bowel	No	11
14	M	46,XY,t(16;16)(p13;q22)7q−	Yes	Mesentery	No	12
41	M	46,XY,inv(16)(p13;q22)	Yes	Small bowel, greater omentum and peritoneum	No	13
58	F	48,XX,inv(16)(p13;q22),+13,+22	Yes	Duodenum	Yes	Current case

MS, myeloid sarcoma; inv(16), inversion of chromosome 16; AML, acute myeloid leukemia; M, male; F, female; NA, not available.
